# Anterolateral thigh flap combined with free fascia lata graft for an adult abdominal wall defect reconstruction: a special case report

**DOI:** 10.3389/fsurg.2025.1679183

**Published:** 2025-10-23

**Authors:** Hang-Chong Shen, Zhong-Xin Sun, Rui-Bin Hu, Dan-Ya Zhou, Tian-Xiang Huang, Xin Wang

**Affiliations:** 1 Department of Hand Microsurgery and Plastic Reconstructive Surgery, Ningbo NO.6 Hospital, Ningbo, China; 2 Ningbo Clinical Research Center for Orthopedics, Sports Medicine & Rehabilitation, Ningbo, Zhejiang, China

**Keywords:** massive abdominal wall defect, reconstructive surgery, congenital giant omphalocele, adult, abdominal wall reconstruction

## Abstract

Large abdominal wall defects pose significant reconstructive challenges. Congenital omphalocele persisting untreated into adulthood is exceptionally rare. We present the first documented case in China of an adult female with a giant congenital omphalocele (defect size: 13 cm × 9 cm; defined as a defect >5 cm in diameter) managed using a novel autologous technique. A combined approach utilizing a free left anterolateral thigh (ALT) with attached fascia lata and an additional harvested fascia lata strip was employed. Preoperative CT confirmed a sternal cleft, rib hypoplasia, scoliosis, and significant rectus diastasis (max 6.8 cm). The ALT flap provided soft tissue coverage, and the attached fascia lata was sutured bilaterally to the anterior rectus sheath. A separate fascia lata strip was interwoven through perforations in the rectus sheaths under tension, reducing diastasis by ∼2.5 cm. Microvascular anastomosis connected the flap pedicle to the inferior epigastric artery. Postoperative recovery was uneventful with stable vital signs, no infection, and excellent flap perfusion. At the 6-month follow-up, the abdominal contour had significantly improved. The flap exhibited moderate thickness without bulkiness, and there was no visceral protrusion in any position. The thigh donor site healed well. The reconstruction achieved sufficient strength, acceptable aesthetics, and preserved fertility potential. The free ALT flap combined with tensioned fascia lata strips represents an effective, autologous solution for complex, large abdominal wall defects, particularly in rare cases such as adult congenital omphalocele, offering robust reconstruction without synthetic materials or secondary surgery.

## Introduction

The abdominal wall is an indispensable protective barrier. The incidence of abdominal wall defects varies; omphalocele occurs in ∼1/4,000–7,000 live births (1), gastroschisis in ∼1/2,000–5,000 (2), and postoperative incisional hernia rates reach 10%–20% (3). Beyond visceral herniation risks, these defects can cause infection, respiratory/circulatory dysfunction, intestinal issues, and adhesive bowel obstruction, presenting a significant clinical challenge (4).

**Figure 1:**
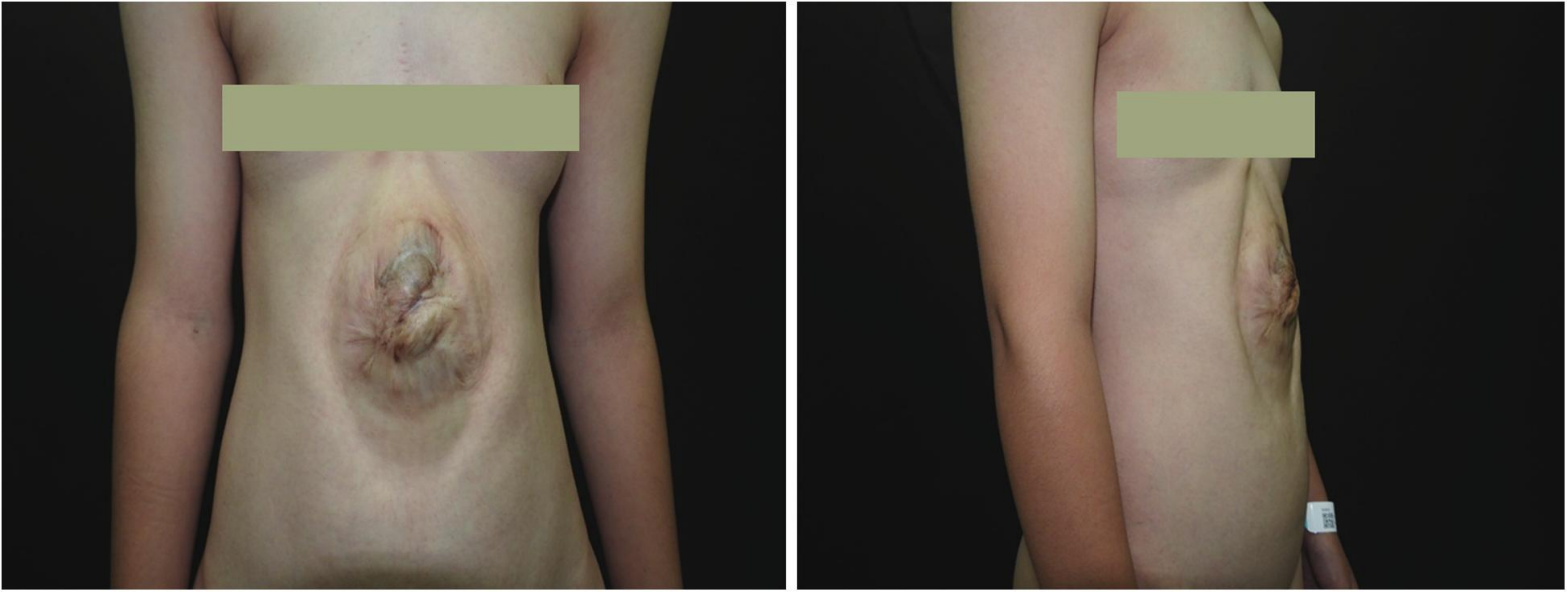
Preoperative photograph of the patient. An elliptical protrusion is visible in the central abdomen. In the standing position, this mass protrudes approximately 2.5 cm above the body surface and measures approximately 13 cm × 9 cm in area. The overlying skin displays significant hyperpigmentation and a wrinkled texture. The umbilicus is absent. The protruding area exhibits visible pulsation that does not correlate with respiratory or cardiac activity.

Management strategies include tension-free mesh repair, component separation, flap reconstruction, tissue expansion, and temporary closure (5–7). Tension-free repair and expansion suit neonates or small defects (8). Adults with extensive loss from trauma, infection, tumor resection, or congenital anomalies often require flap reconstruction. The complexity of repair correlates with defect size. To address large defects, we developed a technique combining a free left anterolateral thigh (ALT) flap with fascia lata strips. This method was successfully applied in a rare case of adult congenital omphalocele (pre-op defect: 13 cm × 9 cm) (Figure 1). Postoperatively, the patient showed stable vitals, improved contour, excellent healing, and no infection. This report details this reconstruction.

## Case description

**Figure 2:**
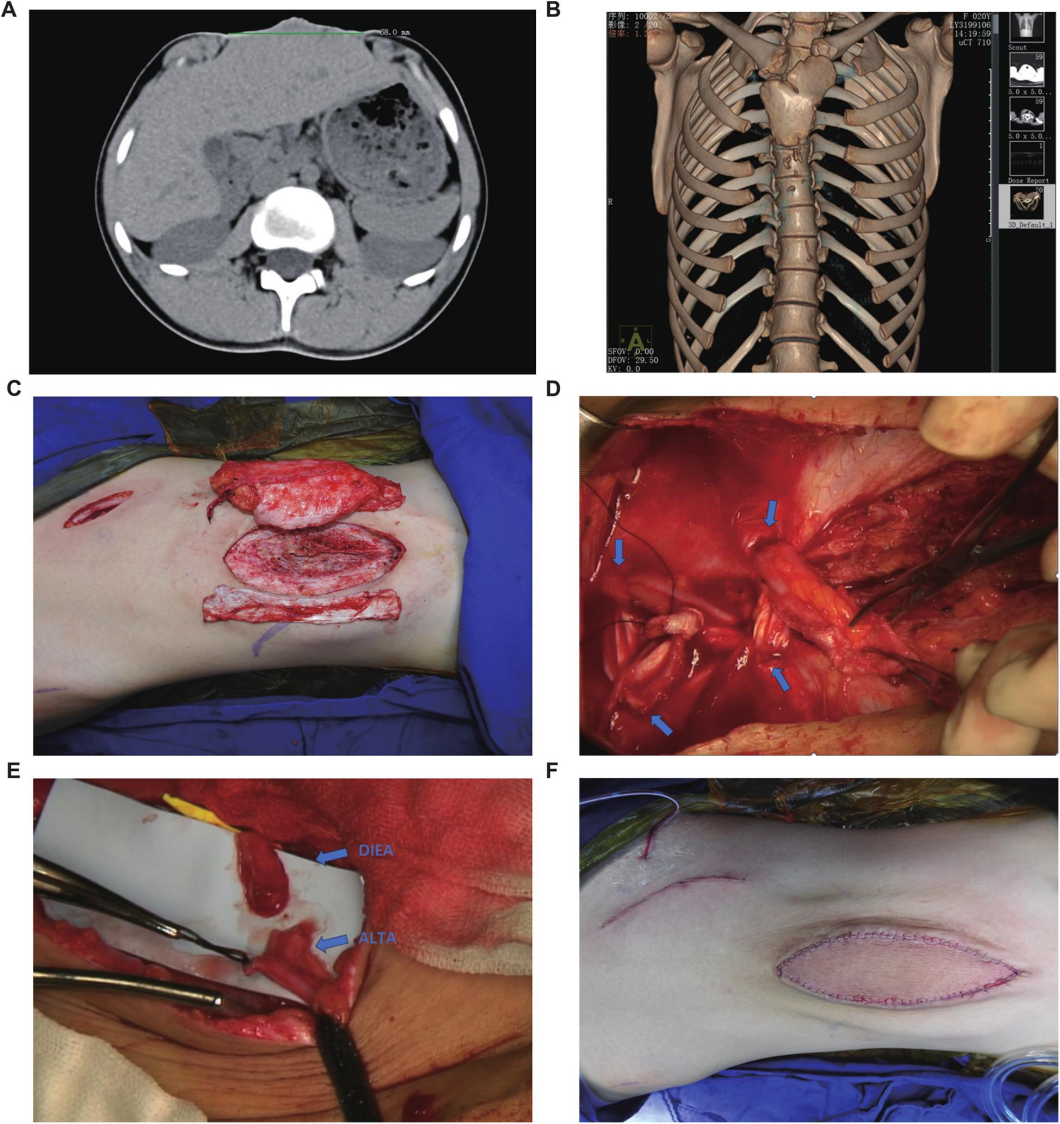
Preoperative examination and surgical procedure. **(A)** Abdominal CT suggests that the maximum diameter of the abdominal defect is 68 mm. **(B)** Chest CT scan (non-contrast) with three-dimensional volume rendering reveals asymmetric bilateral clavicles, sternal cleft with associated rib hypoplasia, and scoliosis. **(C)** The selected autologous free anterolateral thigh flap with fascia lata. **(D)** The inferior end of the rectus abdominis muscle was interwoven with the fascia lata. The blue arrow indicates the fenestration site in the rectus sheath. **(E)** The anastomosis site between the perforating branch of the deep inferior epigastric artery (DIEA) and the perforating branch of the anterolateral thigh artery (ALTAP). **(F)** Postoperative abdominal photograph.

Preoperative non-contrast CT with 3D reconstruction revealed sternal cleft, rib hypoplasia, scoliosis, and diastasis recti (max width 6.8 cm), without organ, cardiopulmonary, diaphragmatic hernia, cardiac malformation, or gastroschisis abnormalities (Figures 2A,B). Following evaluation, congenital omphalocele repair was performed using a left free ALT flap with fascia lata strips. In designing the flap, the following aspects were considered: (1) the abdominal wall structure is similar to that of the lateral thigh, both consisting of a dense fascial layer, an adipose layer, and a cutaneous layer. The fascia lata is anatomically analogous to the abdominal wall fascia. (2) The perforators of the anterolateral thigh vessels have consistent anatomy (as confirmed by ultrasound localization) and a large internal diameter, which relatively reduces the risk of vascular complications. (3) The soft tissue thickness of the abdominal wall and the anterolateral thigh is comparable, minimizing the likelihood of flap bulkiness and potentially avoiding the need for secondary debulking procedures. Regarding the selection of recipient vessels, although the defect was located relatively cranially and the internal thoracic artery and vein could have been feasible options, we ultimately chose the inferior epigastric vascular pedicle for microvascular anastomosis for the following reasons: (1) the internal thoracic artery originates from the subclavian artery and traverses the thoracic cavity to enter the abdominal wall, and its terminal branches contribute to the blood supply of the rectus sheath and abdominal wall. While the diameter of the abdominal segment of the internal thoracic artery is similar to that of the anterolateral thigh artery, utilizing the main trunk of the internal thoracic artery as the nourishing vessel for the flap could potentially compromise blood supply to the ipsilateral abdominal wall postoperatively, particularly considering the increased abdominal wall tension after surgery. (2) The main trunk of the inferior epigastric artery (IEA) has a consistent anatomical location, which is more conducive to preoperative ultrasound localization. Therefore, the ALT flap was selected as the donor site, and the IEA trunk was chosen as the recipient vessel (Figure 2C).

Under general anesthesia in the supine position, a weakened abdominal skin area (∼10 cm × 6 cm) was marked for resection. A left ALT flap (10 cm × 6 cm) with adjacent fascia lata (10 cm × 6 cm) was designed using Doppler-localized perforators. Incision along the flap's lateral border exposed the fascia lata. A dominant intermuscular septal perforator was identified and meticulously dissected retrograde to its source artery with needle-tip electrocautery, ligating branches. The robustly perfused flap was elevated and temporarily secured.

Abdominal skin incision revealed dense peritoneal adhesions. Skin excision exposed the peritoneum. Circumferential subcutaneous dissection (∼5 cm) exposed significantly diastased recti (widest separation ∼5 cm). A preoperative Doppler-guided oblique right lower abdominal incision exposed the right IEA perforator. The main IEA trunk was dissected (∼10 cm to wound), and a subcutaneous tunnel was created.

Returning to the thigh, the ALT flap pedicle was fully exposed, and the anterolateral femoral cutaneous nerve was isolated and transected. After ligating pedicle branches, the flap was elevated with an ∼8 cm pedicle. An additional fascia lata strip (∼10 cm × 4 cm) was harvested. The thigh donor site was closed in layers.

**Figure 3:**
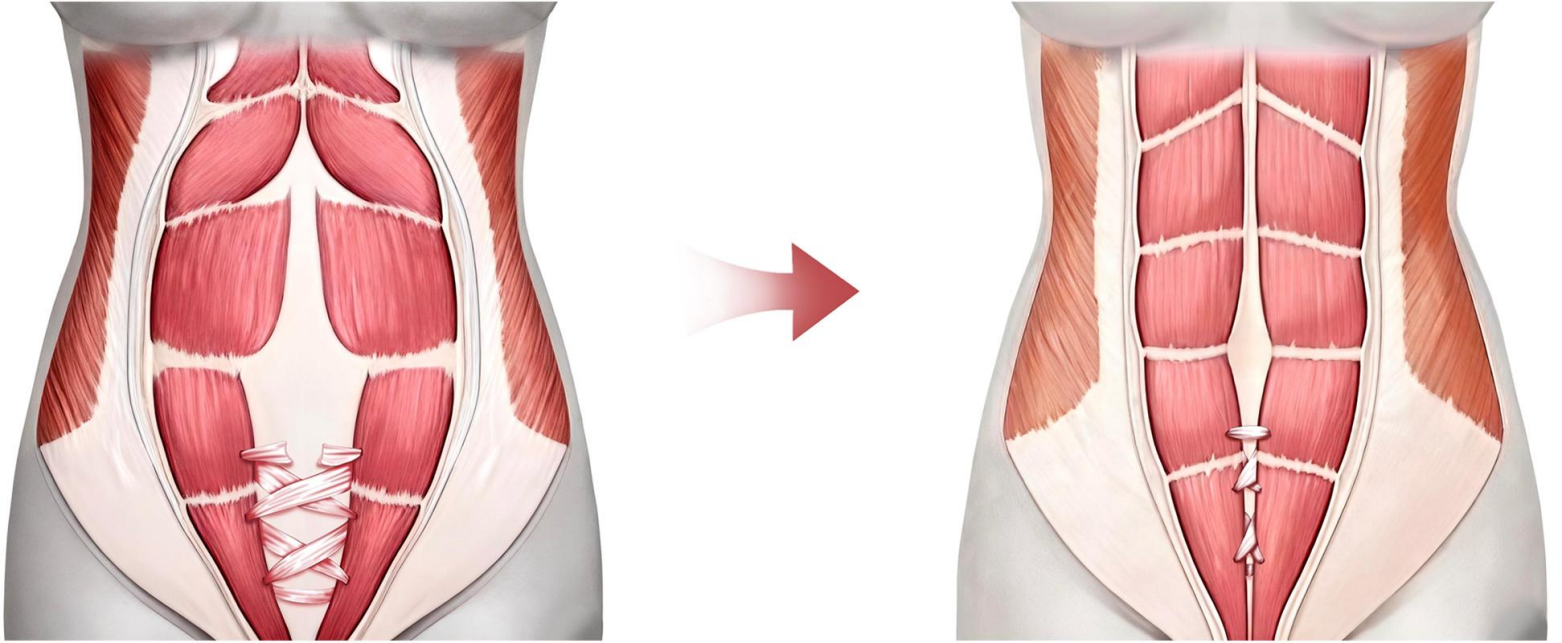
Schematic illustration of the perforated and interlaced weaving technique of the fascia lata strip through the anterior rectus sheath. Three surgical fenestrations were created bilaterally in the inferior portion of the anterior rectus sheath, followed by an interwoven suture technique using a fascia lata strip.

At the abdomen, bilateral anterior rectus sheath perforations were made. The fascia lata strip was anchored to the left rectus, threaded through perforations, tensioned to reduce diastasis by ∼2.5 cm, and secured (Figure 2D). The upper recti were temporarily approximated, reducing diastasis to 2.5 cm. It is important to note that the significant volume of abdominal contents and the resulting high wall tension preclude primary complete approximation of the rectus abdominis muscles, as this would lead to vascular compromise. The ALT flap with attached fascia was transferred; its fascia lata was overlapped bilaterally (∼2 cm) onto the upper diastasis and sutured to the anterior sheath. Removal of temporary sutures confirmed uniform tension before flap inset (Figure 3).

The vascular pedicle was transposed through the tunnel to the IEA trunk. After distal ligation and proximal division, end-to-end microanastomosis (artery and veins) to the IEA trunk was performed under microscopy using 9–0 sutures (Figure 2E). Upon clamp release, robust perfusion was confirmed. Abdominal closure was completed with layered sutures, and closed-suction drains were placed.

**Figure 4:**
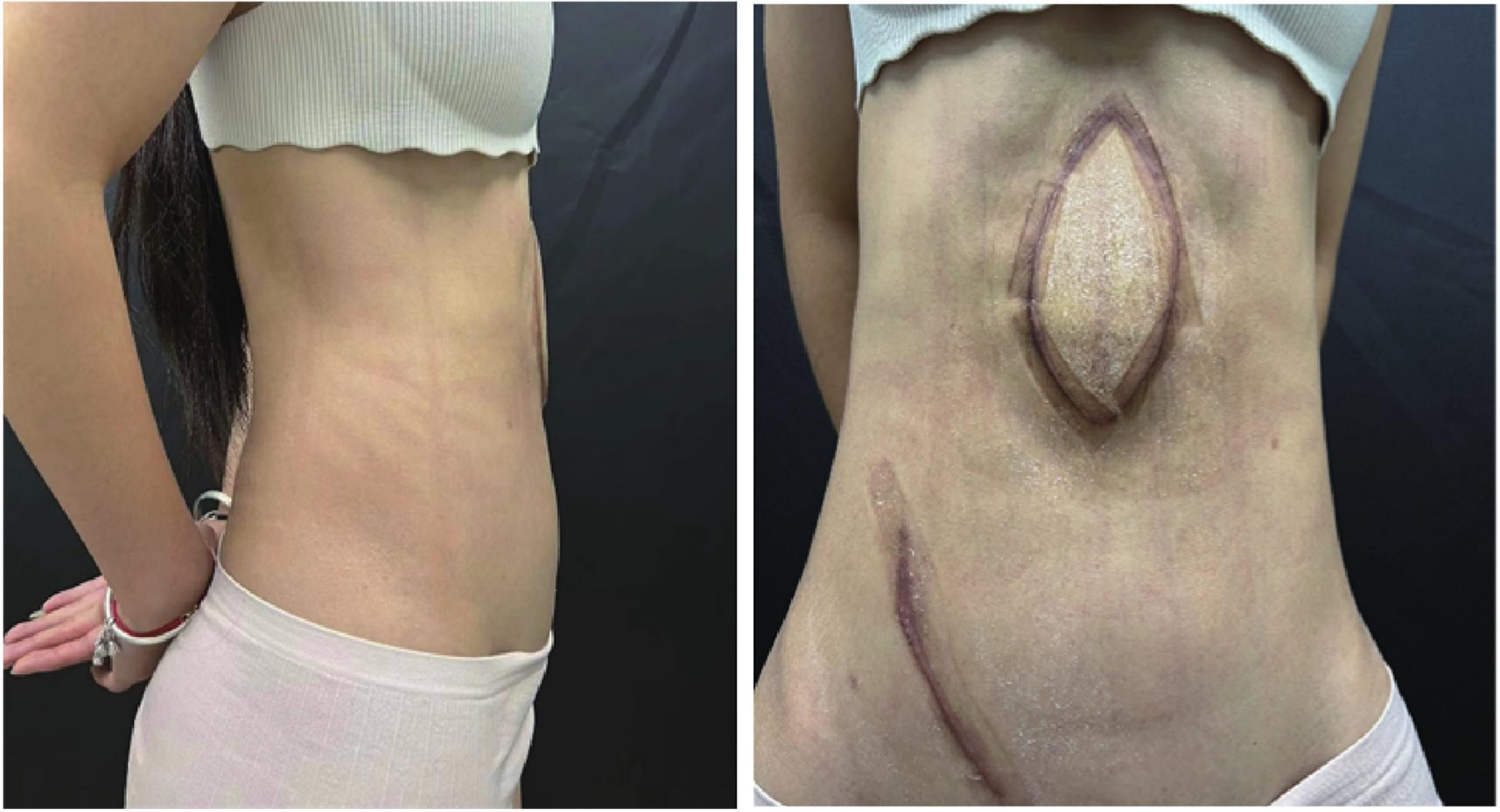
Six-month postoperative abdominal appearance. Postoperative photo at 6-month follow-up demonstrates significant improvement in abdominal contour compared with the preoperative state. The flap shows no significant bulkiness with well-matched soft tissue thickness. Absence of visceral protrusion in the anterior abdominal wall was noted in both standing and prone positions.

The patient returned for a follow-up examination 6 months postoperatively, which showed a significant improvement in abdominal contour (Figure 4). The flap showed moderate thickness without bulkiness, with no visceral protrusion, standing or prone. The thigh donor site healed well, though long-term anti-scar therapy was planned for keloid tendency. The patient exhibited grade V abdominal muscle strength and satisfactory pulmonary function and reported a high quality of life. Furthermore, we conducted follow-up for up to 2 years, and no recurrence of abdominal content protrusion was observed during this period.

## Discussion

This study describes using a combined free ALT flap with fascia lata strips to reconstruct large abdominal wall defects from congenital omphalocele in an adult, achieving acceptable outcomes without secondary surgery. This innovative approach involved careful flap selection. Historical options included ilioinguinal, psoas major, or latissimus dorsi flaps (pedicled/free) (9, 10). Compared to the latissimus dorsi flap, the ALT flap offers several advantages: easier harvest without the need for patient repositioning, more consistent perforator anatomy, a thickness comparable to the native abdominal wall, and superior donor site morbidity. In contrast, the tensor fasciae latae flap is a less conventional option. Its vascular pedicle is less reliably located than that of the ALT flap, and its identification necessitates a higher degree of operator skill and experience with preoperative ultrasound imaging. Conventional primary repair techniques are typically reserved for small abdominal wall defects in pediatric patients or adults. Mesh prostheses are the standard for hernia repair; however, to our knowledge, there are no reported cases in China of using a custom-made mesh for the reconstruction of massive midline abdominal wall defects in adults, and the structural integrity of such custom meshes remains questionable. Both primary repair and mesh techniques only partially restore the parietal peritoneal barrier function and fail to re-approximate the divaricated rectus abdominis muscles, resulting in a significantly higher risk of recurrence. The ALT flap was chosen for its advantages: consistent vascular anatomy, large-caliber perforators, abundant tissue volume, primary donor site closure, minimal morbidity, and lower necrosis risk. Fascia lata strength and texture approximate parietal peritoneum, while ALT soft tissue resembles the abdominal wall. Studies have elucidated the comprehensive mechanical properties and microstructure of human fascia lata, providing a critical foundation for its clinical application (11). Furthermore, comparative research with acellular dermal matrices has been conducted to evaluate their efficacy in facial reanimation surgery. The findings indicate that both fascia lata and ADM alternatives demonstrate satisfactory outcomes in restoring static symmetry and functional oral competence (12, 13). Consequently, fascia lata serves as an excellent autologous material option for abdominal wall reconstruction. Critically, autologous tissue transplantation carries significantly lower rejection risk than synthetic materials, making the free ALT flap with fascia lata ideal (14).

This report details reconstruction in a young, nulliparous woman; preserving future reproductive potential was paramount. The abdominal wall provides mechanical support/protection during pregnancy, facilitates labor, and maintains respiratory/motor function. Our goals were sufficient strength, acceptable aesthetics, preserved fertility potential, and adequate distensibility for future pregnancy volume increases. In collaboration with OB/GYN specialists, we performed an inferior rectus sheath perforation procedure followed by interwoven suturing with fascia lata strips. This technique was employed to enhance the tensile strength of the lower abdominal wall while preserving significant extensibility in the upper abdominal segment under increased intra-abdominal pressure. The management of this case was based on a thorough multidisciplinary discussion. Given the patient's and family's strong desire for future pregnancy—though the patient was not currently eligible for gestation—the primary goal of the intervention was to reinforce the abdominal wall, thereby reducing the risk of abdominal trauma and improving safety during potential future pregnancy. Due to the rarity of such cases, long-term follow-up is required to further evaluate gestational safety outcomes.

Congenital omphalocele requires early intervention (15). Small, uncomplicated defects may be managed conservatively with sterile dressings for spontaneous closure or primary fascial repair. Large defects or severe deficiencies require staged treatment, initially using synthetic materials followed by delayed repair (16). Surgical repair techniques include staged repair with biological mesh and tissue expansion technology. Staged biological mesh repair utilizes medical patches fabricated from biodegradable materials featuring a specific multilayered structure with controlled degradation profiles for each layer (17). Although biological meshes are considered superior to synthetic non-absorbable meshes, conclusive evidence regarding their efficacy remains lacking (18). Tissue expansion technology is a surgical reconstruction technique based on the implantation of an inflatable silicone tissue expander adjacent to the target area beneath the skin or muscle (19). This device is progressively inflated through periodic injections of sterile saline, leading to controlled expansion. The process generates continuous, gentle tension on the overlying skin and soft tissues, resulting in tissue stretching and thinning while simultaneously stimulating epidermal cell proliferation and neotissue generation (20). However, this method is associated with prolonged treatment duration, elevated surgical costs, and reduced quality of life during the therapeutic period. Furthermore, it carries an increased risk of abdominal wall infection, particularly in cases of suboptimal wound care (21). Survival to adulthood untreated is exceptionally rare, with only sporadic global reports. To our knowledge, this is the first documented adult congenital omphalocele case in China. Our patient had a giant omphalocele (defect >5 cm) untreated since birth. China is a developing country, and the patient, located in a remote rural area with limited access to healthcare, did not receive prompt medical attention after birth. The limited self-repair capacity in adulthood significantly increased surgical complexity. Our team successfully reconstructed the defect using this novel approach. Follow-up confirmed no visceral protrusion and satisfactory outcomes, validating the technique's potential for large-scale abdominal wall reconstruction.

In summary, the free ALT flap, with its abundant tissue, reliable pedicle, and robust fascia lata, represents an effective option for abdominal wall reconstruction in adults with large, complex defects from trauma, infection, tumor resection, or congenital malformations.

## Data Availability

The original contributions presented in the study are included in the article/Supplementary Material, further inquiries can be directed to the corresponding author.
